# Lack of HPV in pterygium with no evidence of autoinoculation and the role of cytokines in pterygium with dry eye

**DOI:** 10.1038/s41598-021-82114-6

**Published:** 2021-02-02

**Authors:** Lita Uthaithammarat, Ngamjit Kasetsuwan, Yuda Chongpison, Pimpetch Kasetsuwan, Usanee Reinprayoon, Pornjarim Nilyanimit, Yong Poovorawan

**Affiliations:** 1 Department of Ophthalmology, Faculty of Medicine, Chulalongkorn University and King Chulalongkorn Memorial Hospital, Bangkok, 10310 Thailand; 2 Center of Excellence for Cornea and Stem Cell Transplantation, Department of Ophthalmology, Faculty of Medicine, Chulalongkorn University, Bangkok, Thailand; 3 Excellence Center for Cornea and Limbal Stem Cell Transplantation, Department of Ophthalmology, King Chulalongkorn Memorial Hospital, Thai Red Cross Society, Bangkok, Thailand; 4 Center of Excellence in Biostatistics, Research Affairs, Faculty of Medicine, Chulalongkorn University, Bangkok, Thailand; 5 Faculty of Medicine, Chulalongkorn University, Bangkok, Thailand; 6 Center of Excellence in Clinical Virology, Faculty of Medicine, Chulalongkorn University, Bangkok, Thailand

**Keywords:** Corneal diseases, Human papilloma virus

## Abstract

This study evaluated human papillomavirus’s (HPV) role in pterygium pathogenesis, its autoinoculation from genitalia to ocular surface, potential cytokines involved, and crosstalk cytokines between pterygium and dry eye (DE). This cross-sectional study enrolled 25 healthy controls (HCs) and 116 pterygium patients. Four subgroups of pterygium and DE were used in cytokine evaluations. Conjunctival and pterygium swabs and first-void urine samples (i.e., genitalia samples) were collected for HPV DNA detection using real-time polymerase chain reaction. Tear cytokines interleukin (IL)-6, IL-18, and vascular endothelial growth factor (VEGF) in tears were evaluated. No HPV DNA was detected in conjunctival or pterygium swabs. No association was found between HPV DNA in urine samples and that from conjunctival or pterygium swabs. Tear VEGF levels were significantly higher in pterygium patients than in HCs, with no markedly different levels between primary and recurrent pterygia. Tear IL-6, IL-18, and tear VEGF were significantly higher in participants with DE, regardless of pterygium status. In conclusion, HPV infection was not a pathogenic factor of pterygia. The hypothesis of HPV transmitting from the genitals to ocular surfaces was nullified. Tear VEGF was involved in both pterygia and DE, whereas tear IL-6 and IL-18 played roles only in DE.

## Introduction

Pterygia are degenerative fibrovascular lesions originating from the bulbar conjunctiva and invading the cornea. Pterygia can affect vision inclusively by occupying the visual axis and inducing irregular astigmatism^1^. Several pathological processes may cause pterygia, such as viral infections, inflammation, and genetic predisposition, but the exact pathology remains unclear^2^. Human papillomavirus (HPV) infections are reported to be a pathogenic factor leading to pterygia^3^; however, the prevalence is varied and unpredictable. Few studies have focused on the association between HPV infections and recurrent pterygia, and the results regarding this association are controversial^3–7^.

HPV is a non-enveloped virus, with double-stranded circular DNA. It commonly causes infections in the mucous membranes of the anogenital area and oropharynxx^3^. Several studies have reported the involvement of HPV infections, an increased recurrence risk rate, and more aggressive ophthalmic pterygia^3,8^. Moreover, transmission of genital HPV infections via contaminated fingers is thought to be strongly associated with ocular HPV infections^3,9^. However, this hypothesis remains questionable and controversial^10^. Cervical pap smears are the gold standard for detecting HPV infections from the female genital tract. However, many studies have confirmed the effectiveness and accuracy of using urine instead of cervical pap smears to detect HPV infections from both male and female genitalia^11,12^. The advantages of using urine samples in HPV testing include convenience and the ability to detect HPV DNA from the male genital tract.

Because of the uncertainty of the pterygium pathogenesis, a review analyzed several cytokines involved in pterygium fibrovascular growth, especially vascular endothelial growth factor (VEGF), interleukin (IL)-6, and IL-18^13,14^. The relationship of these cytokines with the pterygium pathogenesis is interesting.

Dry eye disease (DED) is a chronic inflammatory ocular surface disease with various symptoms, including ocular irritation, redness, and epiphora. Inflammatory factors in DED include increased secretion of inflammatory cytokines, such as IL-1α, IL-1β, IL-6 and VEGF, leading to tear hyperosmolarity and conjunctival goblet cell loss, which, in turn, produces more proinflammatory cytokines, creating a vicious cycle^15,16^. Many studies have shown that pterygia and DED are strongly associated and have reported a possible overlap of relevant inflammatory cytokines^17–19^.

This study was conducted to evaluate the HPV infection prevalence in primary and recurrent pterygia and determined whether autoinoculation enables transmitting HPV from the genital tract to the ocular surface. We also evaluated the association between fleshiness and pterygium extension and compared the levels of cytokines, including IL-6, IL-18 and VEGF, in the tears of primary pterygium patients, recurrent pterygium patients and participants without ocular disease. These cytokines were compared among different grades and stages of pterygia and evaluated regarding pterygia and dry eye (DE) status.

## Results

This study included 141 participants: 25 healthy controls (HCs) and 116 pterygium patients. No participants had ever received an HPV vaccine or had histories of cervical cancer or cervical cell abnormalities. Table 1 shows the participant demographics. An insignificant difference was noted in the mean age between the subgroups of the HCs, primary pterygium patients, and recurrent pterygium patients (*p* = 0.341) and between the HCs and pterygium patients (*p* = 0.385).

**Table 1:** Patient demographic and clinical characteristics.

**Total number of patients (n)**	
Male	50 (35.5%)
Female	91 (64.5%)
**Side (n)**	
Right	73 (51.8%)
Left	68 (48.2%)
**Age (years ± SD)**	
HC	54.16 ± 10.62
Primary	56.17 ± 13.92
Recurrent	60.5 ± 15.25
**Subgroups (n)**	
HC	25 (17.7%)
Primary pterygium	100 (70.9%)
Recurrent pterygium	16 (11.4%)
**Grade (n)**	
T1	22 (19%)
T2	36 (31%)
T3	58 (50%)
**Stage (n)**	
I + II	23 (19.8%)
III	85 (73.3%)
IV	8 (6.9%)
**% Dry eye**	
HC	44% (11/25)
Pterygium (both primary and recurrent)	41.4% (48/116)

HC = healthy control; grade of pterygium according to fleshy and translucency by Tan et al. classification; stage of pterygium according to extension of pterygium by Johnston, Williams, and Sheppard classification system.

### HPV detection

No HPV DNA was detected in the conjunctival swabs of either the HCs or pterygium patients via real-time PCR. Conversely, HPV DNA was detected in 17 (12.06%) urine samples: 6 from the HCs, 9 from the primary pterygium patients, and 2 from the recurrent pterygium patients. HPV genotypes 16, 35, 42, 43, 45, 51, 52, 54, 58, 66, 68, 70, and 82 were detected in these samples, with genotype 52 being the most common in 5 of the urine samples.

McNemar’s test revealed that the percentage of HPV detected in the urine samples (12.06%) differed significantly from that in the conjunctival (0%) and pterygium (0%) swabs (*p* < 0.001).

### Pterygium

A moderately strong correlation was found between pterygium grade and stage, with a Spearman correlation coefficient of 0.78.

#### Tear cytokine assessment

To compare cytokine concentrations, 141 participants were divided into 25 HCs, 100 primary pterygium patients, and 16 recurrent pterygium patients. The pterygium patients were further combined into one group to compare the cytokines between the 25 HCs and 116 pterygium patients.

**Figure 1:**
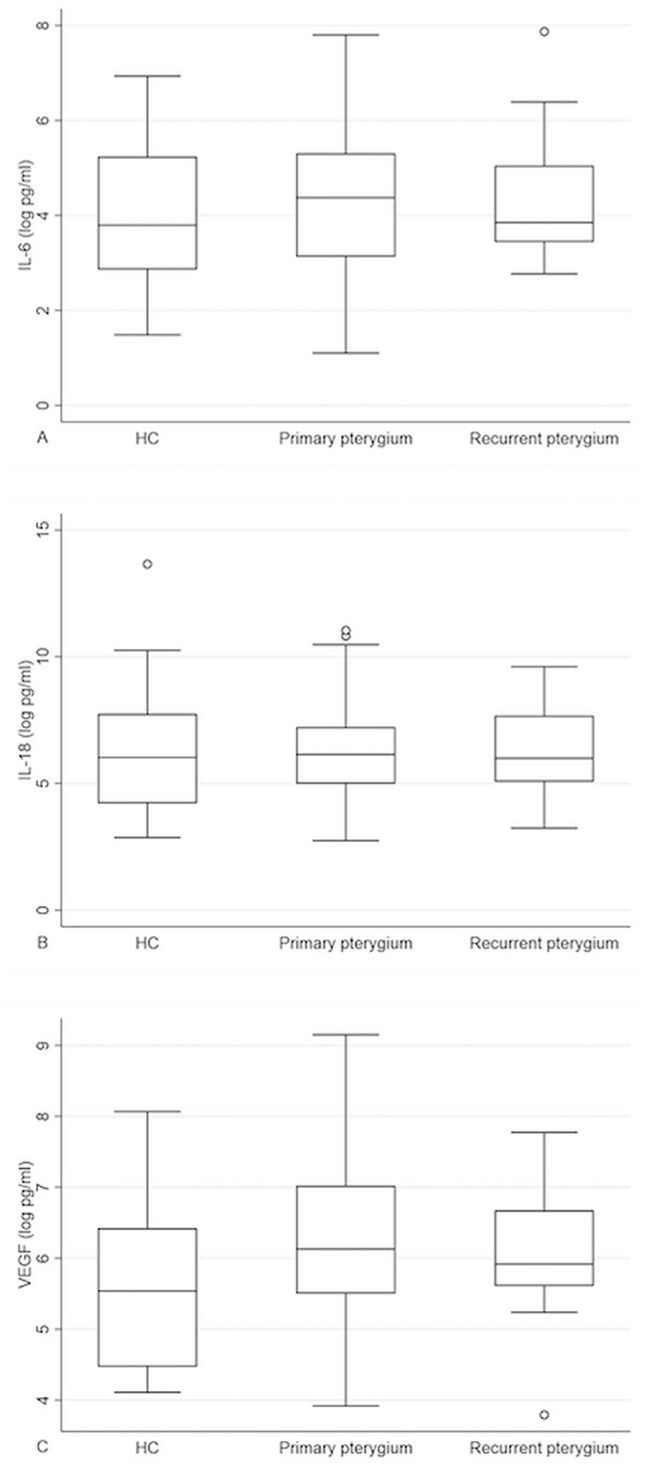
Boxplot of tear cytokine concentration (after logarithmic transformation) in healthy control, primary pterygium, and recurrent pterygium groups. (**A**) Tear IL-6 concentration in each group; (**B**) Tear IL-18 concentration in each group; (**C**) Tear VEGF concentration in each group.

Table 2 shows the geometric mean values of all tear cytokine concentrations. Subgroup analysis showed no statistically significant differences in tear IL-6, IL-18, or VEGF concentrations among the HCs, primary pterygium patients, and recurrent pterygium patients (*p* = 0.662, 0.938, and 0.081, respectively; Fig. 1). The tear cytokine concentrations in the primary pterygium patients were similar to those of the recurrent pterygium patients.

**Table 2:** Tear cytokine concentration in each group of participants.

Cytokines	Geometric mean (%CV)
**IL-6 tear film level (pg/ml)**	
HC	55.52 (298.24)
Pterygium (primary + recurrent)	73.79 (255.72)
Primary	73.14 (260.95)
Recurrent	78.00 (241.09)
**IL-18 tear film level (pg/ml)**	
HC	603.99 (2944.97)
Pterygium (primary + recurrent)	517.11 (559.66)
Primary	513.91 (551.96)
Recurrent	537.39 (689.58)
**VEGF tear film level (pg/ml)**	
HC	246.20 (150.79)
Pterygium (primary + recurrent)	484.45 (156.23)
Primary	497.27 (157.54)
Recurrent	407.71 (157.77)

%CV = percent coefficient of variation; IL = interleukin; HC = healthy control; VEGF = vascular endothelial growth factor.

**Figure 2:**
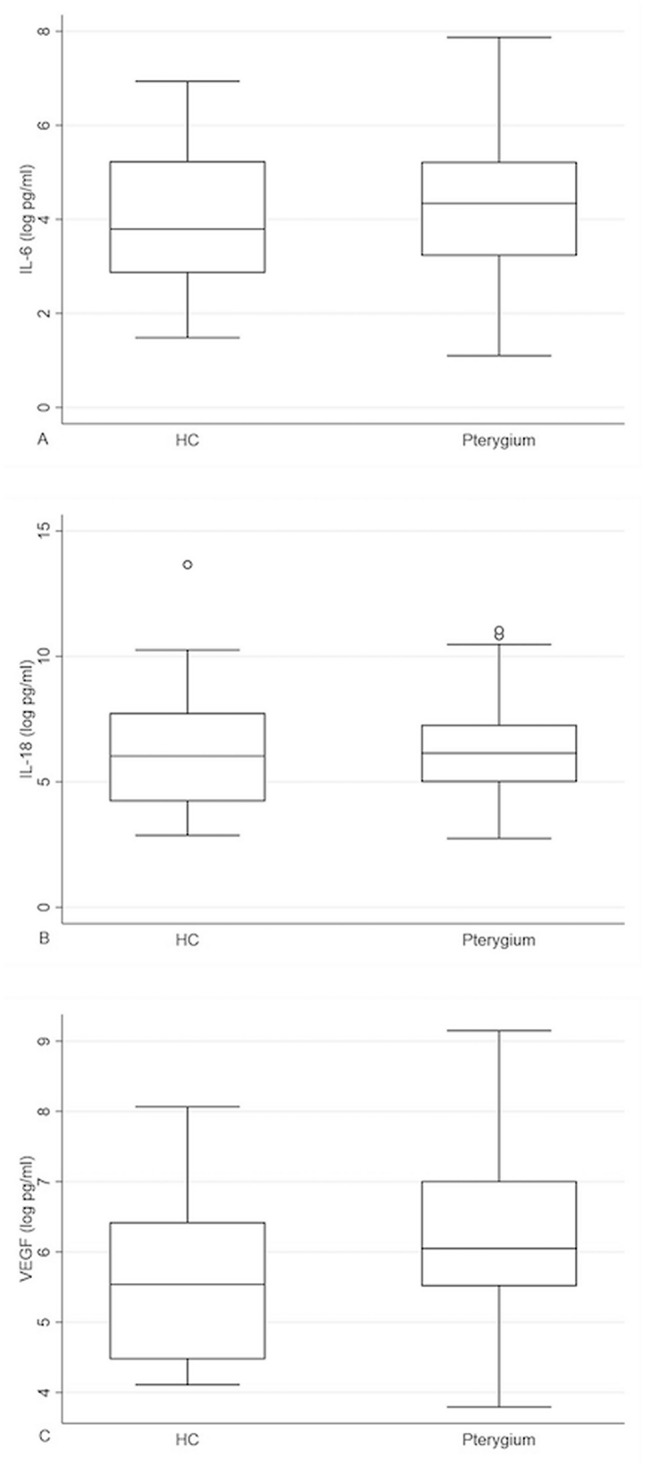
Boxplot of tear cytokine concentration (after logarithmic transformation) in healthy control, and pterygium groups. (**A**) Tear IL-6 concentration in each group; (**B**) Tear IL-18 concentration in each group; (**C**) Tear VEGF concentration in each group with a significantly higher concentration of VEGF in pterygium group.

The geometric mean of the tear VEGF concentration in the pterygium group was 1.97 times higher (95% CI 1.07–3.60) than that in the HCs (*p* = 0.029; Fig. 2). However, differences in tear IL-6 and IL-18 concentrations were insignificant between the HCs and pterygium patients (*p* = 0.371 and *p* = 0.727, respectively).

Tear IL-6, IL-18, and VEGF concentrations did not significantly differ among the grades (*p* = 0.377, *p* = 0.421, and *p* = 0.294, respectively) or stages (*p* = 0.166, *p* = 0.850, and *p* = 0.627, respectively).

#### Correlation between tear cytokine concentrations and Schirmer I test results

**Figure 3:**
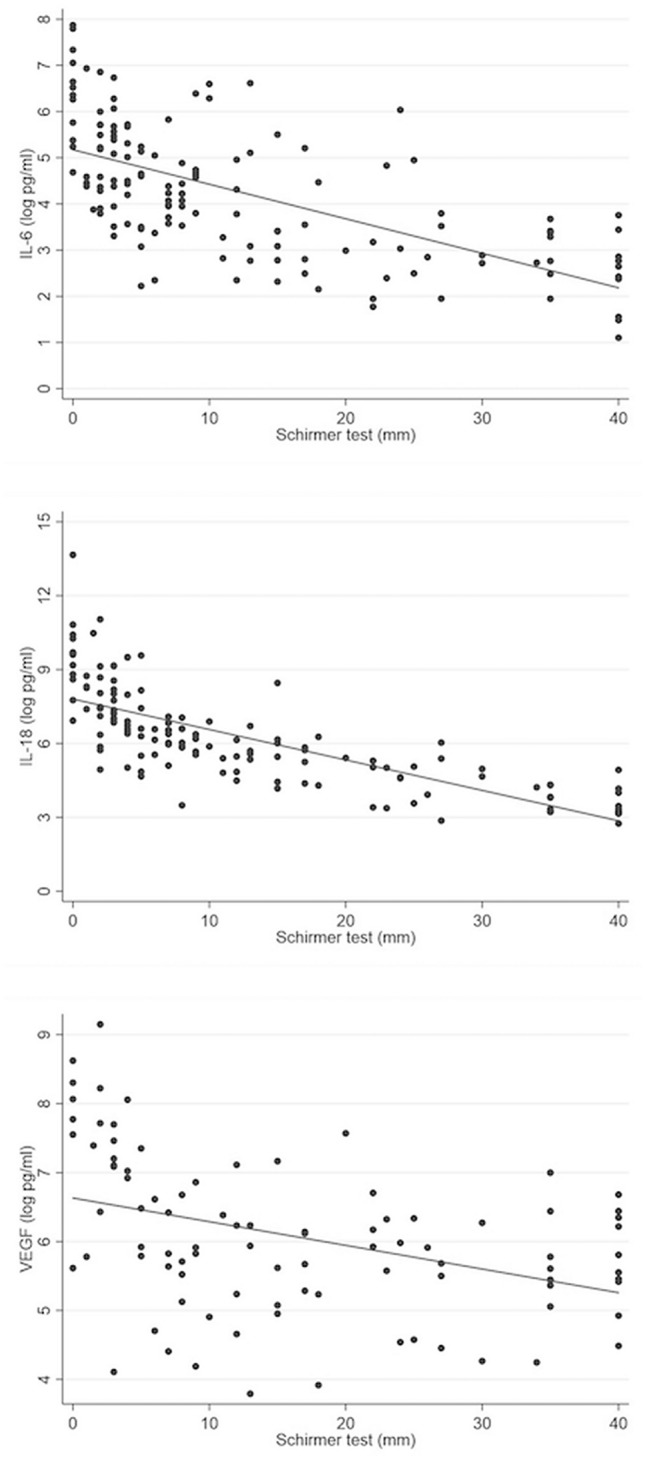
Scatter plot of correlation between Schirmer test and concentration of tear cytokine (after logarithmic transformation). (**A**) Correlation between Schirmer test (mm) and concentration of IL-6 (log pg/ml); (**B**) Correlation between Schirmer test (mm) and concentration of IL-18 (log pg/ml); (**C**) Correlation between Schirmer test (mm) and concentration of VEGF (log pg/ml).

Analyzing the relationship between tear production on the Schirmer I test and tear cytokine concentrations (after logarithmic transformation; Fig. 3) revealed moderately strong negative correlations in IL-6 and IL-18, with correlation coefficients of -0.638, and -0.756, respectively. However, VEGF showed a weak negative correlation (-0.399). Hence, the Schirmer I test, an indicator of DE, was a confounding factor for cytokine evaluation and was corrected in the binary logistic regression and used to categorize DE patients in further subgroup analyses.

#### Binary logistic regression analysis

Binary logistic regression controlling for the Schirmer I test as a confounding factor, showed that the VEGF covariate differed significantly between the HC and pterygium groups (adjusted OR = 1.89; 95% CI = 1.07–3.33; *p* = 0.028). However, no significant differences were found in the IL-6 (adjusted OR = 1.26; 95% CI = 0.84–1.90) or IL-18 (adjusted OR = 0.91; 95% CI = 0.66–1.25) covariates.

#### Subgroup analysis considering DE in HCs and pterygium patients

Because the cytokine concentrations were similar between the primary and recurrent pterygium groups, we also compared the cytokine concentrations in the pterygium group and the HCs relative to DE status. Table 3 shows the tear cytokine concentrations in each group and the p-values from the linear regression analysis.

**Table 3:** Geometric mean of tear cytokine concentration in subgroup analysis considering dry eye status.

	HC-DE (n = 14)	HC + DE (n = 11)	Pterygium-DE (n = 68)	Pterygium + DE (n = 48)	Linear regression, p
IL-6 (%CV) (pg/ml)	21.27 (119.53)	188.32 (176.75)	40.91 (195.37)	170.18 (184.84)	< 0.001
IL-18 (%CV) (pg/ml)	144.19 (370.01)	3739.23 (2237.33)	169.35 (163.63)	2514.07 (298.12)	< 0.001
VEGF (%CV) (pg/ml)	181.81 (94.62)	479.72 (258.66)	313.85 (98.27)	1633.50 (111.82)	< 0.001

HC-DE = healthy control without dry eye; HC + DE = healthy control without dry eye; Pterygium-DE = pterygium participants without dry eye; Pterygium + DE = pterygium participants with dry eye; IL = interleukin; VEGF = vascular endothelial growth factor; %CV = percent coefficient of variation.

#### IL-6

The geometric mean of the IL-6 concentration in the HCs with DE was 8.86 times higher (95% CI 2.49–31.50) than that of the HCs without DE (*p* < 0.001) and 4.60 times higher (95% CI 2.11–10.02) than the pterygium groups without DE (*p* = 0.001). The geometric mean of the IL-6 concentrations of pterygium patients with DE was 8 times higher (95% CI 3.07–20.83) than that of the HCs without DE (*p* < 0.001) and 4.16 times higher (95% CI 2.30–7.53) than that of the pterygium patients without DE (*p* < 0.001).

#### IL-18

The geometric mean of the tear IL-18 concentrations in the HCs with DE was 25.93 times higher (95% CI 5.62–119.76) than that of those without DE (*p* < 0.001) and 22.08 times higher (95% CI 8.64–56.43) than that of the pterygium groups without DE (*p* < 0.001). The geometric mean of the IL-18 concentrations in the pterygium patients with DE was 17.44 times higher (95% CI 5.50–55.25) than that of the HCs without DE (*p* < 0.001) and 14.85 times higher (95% CI 7.26–30.37) than that of the pterygium groups without DE (*p* < 0.001).

#### VEGF

The geometric means of the tear VEGF concentrations in pterygium patients with DE were 8.98 times (95% CI 3.81–21.20), 3.41 times (95% CI 1.09–10.69), and 5.20 times (95% CI 2.87–9.44) higher than those of the VEGF concentrations in HCs without DE (*p* < 0.001), HCs with DE (*p* = 0.031), and pterygium patients without DE (*p* < 0.001), respectively.

## Discussion

We found no HPV infections in the pterygia or conjunctiva nor any evidence that HPV was transmitted via autoinoculation from the genital tract to the ocular surface. Moreover, the pterygium grade, which was determined from the fleshiness, was moderately to strongly correlated with the pterygium stage based on the extent to which the pterygium invaded the cornea. In the cytokine results, only the tear VEGF concentration in the pterygium group was significantly higher than that of the HCs. Tear IL-6, IL-18, and VEGF concentrations of the primary pterygium patients were very similar to those of the recurrent pterygium patients. In addition, tear IL-6, IL-18, and VEGF were associated with DE status. Therefore, in this study, VEGF was an inflammatory cytokine that was involved in the coexistence of pterygia and DE.

### HPV and pterygium

Many studies have found HPV DNA in pterygium samples using different techniques including PCR, Southern blotting, immunohistochemistry, in situ hybridization, and the Hybrid capture assay II. HPV prevalence varied among these studies, ranging from 0%–100%, and the strains of detected HPV DNA also differed among studies^3,20^. Thus, no consensus has been reached regarding HPV as a possible pathogenic cofactor. Many studies have used exfoliative cytology, which is a swab technique for detecting HPV DNA in pterygia, conjunctival epithelial neoplasia, and normal conjunctiva^5,10,20,21^. Chalkia et al. compared HPV detection from noninvasive swab techniques with that from excised pterygium tissue and showed favorable results, including 88.89% sensitivity, 100% specificity, a 92.31% negative predictive value, and a 100% positive predictive value^20^. Thus, in our study, we used this method and real-time PCR, which was highly sensitive and allowed rapid detection with quantification of viral gene expression. Real-time PCR can eliminate the risk of carryover contamination. Our results showed no HPV DNA in the 116 pterygium swabs, consistent with nine studies that also showed no HPV DNA in pterygia using different HPV detection techniques^3,22^. Moreover, Tulvatana et al. studied HPV prevalence in ocular surface squamous neoplasia in Thailand and found no HPV DNA^23^. Consequently, we believe that HPV is not a pathogenic cofactor causing pterygium. Detection of HPV DNA in pterygia in some studies may have been due to contaminated samples.

Sonnex et al. reported that HPV from genitalia was transmitted via finger-genital contact^9^, and many studies have mentioned this mechanism of autoinoculation between HPV in the genital area and ocular surfaces, including pterygia^3,22,24–26^. However, in this study, HPV in the genital tract was not associated with HPV-infected conjunctiva or pterygia. We found many HPV strains (e.g., 16, 45, 52, 54, 58, 66) in urine samples; these strains have previously been reported in pterygium tissue^3,20^. The pterygium-related strains published in those studies were found in urine samples in this study, but not in the pterygia or conjunctiva. Thus, no evidence was found of HPV transmission via autoinoculation from the genitalia to the pterygium or ocular surface.

### Pterygium and tear cytokine concentrations

This study found a moderate to strong association between pterygium grade and stage. In other words, more aggressive pterygia with more fleshiness were more likely to recur after surgical excision^27,28^. This was correlated with a higher stage being categorized by the length or extension of the pterygium. This was consistent with a previous study of the association between pterygium fleshiness and expansion^29^. Consequently, more extended pterygia will have higher recurrence rates after surgery and require prompt treatment if the progression appears to extend to the center of the cornea.

In our study, the mean VEGF concentration in the pterygium group was significant at 1.97 times higher than that of the HC group, and binary logistic regression controlling for the Schirmer I test as a confounding factor showed that the VEGF covariate differed significantly between the HC and pterygium groups. These results indicated an association between VEGF and pterygia. Moreover, VEGF concentrations did not significantly differ between the primary and recurrent groups. Many studies have reported that VEGF is involved in causing pterygia^13,17,30^. However, few studies have compared VEGF concentrations between primary and recurrent pterygium groups^30^. The role of VEGF as a pathological factor in pterygia is thought to be that increased VEGF expression causes angiogenesis and lymphangiogenesis, which, in turn, might affect the normal metabolism of conjunctival cells and promote them to become pterygia^13^. Regarding cytokines, many studies have depicted the relationship between IL-6 and pterygia. Sun and Chang also described significantly higher IL-18 levels in pterygia compared with those in a normal conjunctiva^13,14^. In contrast, IL-6 and IL-18 concentrations in the pterygium group did not significantly differ from those of the HCs, and binary logistic regression controlling for the Schirmer I test as a confounding factor showed that the IL-6 and IL-18 covariates did not significantly differ between the HC and pterygium groups. Van et al. also reported an insignificant difference in IL-6 between the pterygium and normal conjunctiva groups^30^. Thus, we believe that VEGF is a contributing factor in the pterygium pathogenesis, whereas the roles of IL-6 and IL-18 remain inconclusive.

Our study showed no difference in cytokines by pterygium stage or grade, and Van et al. found no difference in cytokines in corneal areas affected by pterygia^30^.

In our study, IL-6, IL-18, and VEGF concentrations in pterygium patients and HCs with DE were significantly higher at 4.16–8.86, 14.85–25.93, and 3.41–8.98 times higher, respectively, than those of the other groups without DE. A moderate to strong inverse correlation was found between IL-6 and IL-18 concentrations and the Schirmer test, indicating tear secretion associated with DE. IL-6 and IL-18 were highly elevated in participants with DE with or without pterygia in this study. Although the VEGF concentration was significantly elevated in DE participants, VEGF was not associated with the Schirmer test, likely because VEGF is involved in both pterygia and DE. These associations might be due to the following mechanism. IL-6 is involved in DE^15,16^ and is released from the ocular surface epithelium when immunoregulation of the ocular surface is disrupted. This causes epithelial damage and activates antigen-presenting and natural killer cells, which further leads to inflammation in DE^15^. Niu et al. reported on the relationship of IL-18 with DE^31^. They found that IL-18 mRNA expressions in DE associated with and without Sjogren’s syndrome were significantly higher than those of the HCs. IL-18 is secreted by caspase-1 activation of the Nod-like receptor family pyrin domain-containing 3 (NLRP3) inflammasome. IL-18 recruits innate immune responses and regulates the subsequent adaptive immune response. Our study also strongly evidenced the association between IL-18 and DE. However, the exact mechanism of IL-18 in DE remains unclear. Moreover, many studies have reported the coexistence of pterygia and DE^17–19,32^. Theories of DE development in pterygium patients include that the same aggravating factor that causes pterygium development may also induce ocular surface inflammation, including ultraviolet radiation or the pterygium itself causing ocular surface irregularity and consequently a loss of tear film stability. In addition to being involved in angiogenesis and lymphagiogenesis of pterygia, VEGF may also increase vascular permeability. These mechanisms could expand the inflammatory reactions in pterygium tissue and the ocular surface^33^. Liu et al. described that VEGF and MMP-9, which are upregulated by VEGF through the nuclear factor kappa-light-chain-enhancer of activated B cells (NF-kB) signaling pathway, play important roles in the crosstalk between pterygia and DE^15,17,33^. In this study, we also found significantly higher VEGF levels in the pterygium group, which were 1.97, 3.41, and 5.20 times those of the HC group regardless of DE status, in pterygium patients with DE compared with HCs with DE and in pterygium patients with DE compared with pterygium patients without DE, respectively. Although VEGF concentrations in pterygium patients without DE were insignificant at 1.73 times higher than those of HCs without DE, binary logistic regression controlling for the Schirmer I test confirmed a significant association between VEGF and pterygia. Our evidence supports the role of VEGF in the crosstalk between pterygia and DE. In addition, evidence indicating similar tear VEGF levels between primary and recurrent pterygia suggests that anti-VEGF medication could be used to treat both primary and recurrent pterygia.

This study had some limitations. First, we did not use tear film break-up time or corneal staining to evaluate DED because fibrovascular tissue in pterygia that invade the cornea could disrupt the tear film layer and corneal staining, making the assessment of DED based on these two parameters unreliable. Second, we evaluated only fleshiness and extension of the pterygium. Neovascularization and the total area of the pterygium tissue invading the cornea should be observed together with these parameters. However, we primarily used fleshiness and pterygium extension in this study because fleshiness indicated the possibility of pterygium recurrence, and extension of the pterygium invasion to involve the pupil area could affect patients’ vision. These two parameters are strongly related to pterygium management.

## Methods

The institutional review board of the faculty of medicine, Chulalongkorn University, approved this study, which adhered to the tenets of the Declaration of Helsinki. All patients provided informed consent before being enrolled.

### Participants and baseline clinical characteristics

Data were collected from January 2020 to April 2020 at an outpatient clinic of King Chulalongkorn Memorial Hospital. One hundred sixteen pterygium patients and 25 age-matched healthy volunteers were enrolled. The 116 pterygia included 100 primary pterygia and 16 recurrent pterygia. If patients had pterygia in both eyes, the eye with the more severe pterygium grade was included. To ensure representative HCs, the HCs were required to have no history of ocular pathology before entering the study. Exclusion criteria for the HCs are in the supplementary material. We compared cytokine concentrations among HC subgroup for the conjunctiva and primary and recurrent pterygia and between the HC conjunctiva and pterygium groups. Demographic data were recorded for all participants.

### Diagnosis, staging, and pterygium grading

A slit-lamp examination was performed on the HCs to confirm that they had a normal cornea and conjunctiva and for grading and staging in pterygium patients. Pterygia were graded based on the relative translucency of the pterygium tissue according to Tan et al.’s classification system: grade T1: atrophic pterygia with unobscured episcleral vessels; T2: intermediate pterygium with partially obscured episcleral vessels, and T3: fleshy pterygium with obscured episcleral vessels^27^. The pterygium staging was classified by the length of the pterygium tissue invading the limbus and cornea as per the classification system of Johnston, Williams, and Sheppard^34^.

### Tear collection and definition of dry eye

After the slit-lamp examination, the Schirmer I test was performed by placing Schirmer strips (32 K. Supply Co., Ltd., Bangkok, Thailand) at the participant’s inferior fornix without anesthetic eye drops. After 5 min, the wetness of the filter paper was measured in mm from the initial fold. All Schirmer strips were collected in 2-mL centrifuge tubes at 4 °C, then transported at − 20 °C until cytokine analysis. DE status in this study was defined by a Schirmer test result of < 5.5 mm with complaints of ocular symptoms including discomfort, redness, irritation, dryness, and photophobia. After completing the Schirmer test, the conjunctiva and pterygia were swabbed.

### Tear cytokine analysis

The protocol for analyzing the tear cytokines was followed as previously described^35^. To avoid diurnal variations in tear cytokine levels, the Schirmer test was performed from 1 to 3 pm for all participants. All tear cytokines were analyzed using the Bio-Plex 200 system (Bio-Rad, Hercules, CA, USA).

### Ocular and urine sample collection for HPV detection

Each participant was swabbed with a FLOQSwab (Copan Diagnostics Inc., Murrieta, CA, USA) at the pterygium (pterygium group) or the normal conjunctiva (HCs). Participants were also taught how to collect urine in a special urine collection container (Colli-Pee device; Novosanis, Wijnegem, Belgium). DNA extraction methods from the urine and ocular samples are in the supplementary material.

### HPV detection

Extracted DNA from the eye swab and urine samples was subjected to an HPV genotyping assay using Anyplex II HPV28 (Seegene, Seoul, South Korea) as previously reported^36,37^ (Supplementary Material).

### Statistical analysis

Demographics and baseline clinical characteristics were reported as the mean and standard deviation or as the frequency and percentage. Descriptive statistics were conducted using unpaired student’s t-test or Analysis of Variance (ANOVA), whenever it is appropriate. The HPV detection prevalence in the ocular swabs and urine samples was compared via McNemar’s test. The cytokine concentrations were described as geometric means with the percent coefficient of variation (%CV), while normal distribution was determined using histograms and the Shapiro–Wilk test for normality after logarithmic transformation. Linear regression using Tukey’s method was used to evaluate the differences in logarithmic mean values of the cytokine concentrations among various groups based on pterygium grade, stage, and DE. Correlation analysis between pterygium grade and stage was evaluated using Spearman’s Rank correlation coefficient. Correlations between log-transformed cytokine concentrations and the Schirmer test (in mm) were evaluated by Pearson’s correlation. Multivariate logistic regression, presented as an adjusted odds ratio (OR) and 95% confidence interval (CI), was used to assess the effect of cytokine concentrations on presence of pterygia, adjusting for the Schirmer test.

Stata version 15.1 was used for all analyses (StataCorp, 2017; Stata Statistical Software, Release 15; StataCorp, LLC, College Station, TX, USA), and an alpha level of 0.05 was used to determine statistical significance.

## Conclusion

HPV infection was not a pathological factor in pterygium development. We found no evidence that HPV was transmitted via autoinoculation from the genital tract. VEGF was noted as an important factor in the crosstalk between pterygia and DE, whereas IL-6 and IL-18 were solely involved with DE.

## Supplementary Information

Supplementary Information.
