# Author Correction: Lack of HPV in pterygium with no evidence of autoinoculation and the role of cytokines in pterygium with dry eye

**DOI:** 10.1038/s41598-021-89723-1

**Published:** 2021-05-04

**Authors:** Lita Uthaithammarat, Ngamjit Kasetsuwan, Yuda Chongpison, Pimpetch Kasetsuwan, Usanee Reinprayoon, Pornjarim Nilyanimit, Yong Poovorawan

**Affiliations:** 1 Department of Ophthalmology, Faculty of Medicine, Chulalongkorn University and King Chulalongkorn Memorial Hospital, Bangkok, 10310 Thailand; 2 Center of Excellence for Cornea and Stem Cell Transplantation, Department of Ophthalmology, Faculty of Medicine, Chulalongkorn University, Bangkok, Thailand; 3 Excellence Center for Cornea and Limbal Stem Cell Transplantation, Department of Ophthalmology, King Chulalongkorn Memorial Hospital, Thai Red Cross Society, Bangkok, Thailand; 4 Center of Excellence in Biostatistics, Research Afairs, Faculty of Medicine, Chulalongkorn University, Bangkok, Thailand; 5 Faculty of Medicine, Chulalongkorn University, Bangkok, Thailand; 6 Center of Excellence in Clinical Virology, Faculty of Medicine, Chulalongkorn University, Bangkok, Thailand

Correction to: *Scientific Reports* 10.1038/s41598-021-82114-6, published online 02 February 2021

The original version of this Article contained a typographical error in the spelling of the author Yong Poovorawan, which was incorrectly given as Yong Pooworawan. This has now been corrected in the PDF and HTML versions of the Article, and in the accompanying Supplementary Information file.

