# Supplementary material for: Lack of HPV in pterygium with no evidence of autoinoculation and the role of cytokines in pterygium with dry eye
Source: Sci Rep. 2021 Feb 2;11:2842. doi: 10.1038/s41598-021-82114-6 (PMC7854664; doi:10.1038/s41598-021-82114-6)
Supplement: Supplementary file 1 — Supplementary Information. [file 41598_2021_82114_MOESM1_ESM.doc]

**Lack of HPV in pterygium with no evidence of autoinoculation and the role of cytokines in pterygium with dry eye**

Lita Uthaithammarat^1^, Ngamjit Kasetsuwan^1,2*^, Yuda Chongpison^3^, Pimpetch Kasetsuwan^4^, Usanee Reinprayoon^1,2^, Pornjarim Nilyanimit^5^, Yong Poovorawan^5^

^1^Department of Ophthalmology, Faculty of Medicine, Chulalongkorn University and King Chulalongkorn Memorial Hospital, Bangkok, Thailand.

^2^Center of Excellence for cornea and stem cell transplantation, Department of Ophthalmology, Faculty of Medicine, Chulalongkorn University and Excellence center for cornea and limbal stem cell transplantation, Department of Ophthalmology, King Chulalongkorn Memorial Hospital, Thai Red Cross Society, Bangkok, Thailand.

^3^Center of Excellence in Biostatistics, Research Affairs, Faculty of Medicine, Chulalongkorn University, Bangkok, Thailand.

^4^Faculty of Medicine, Chulalongkorn University.

^5^Center of Excellence in Clinical Virology, Faculty of Medicine, Chulalongkorn University, Bangkok, Thailand.

*Corresponding author: Ngamjit Kasetsuwan, MD Department of Ophthalmology, Faculty of Medicine, Chulalongkorn University, Bangkok, Thailand 10310, Email address: [ngamjitk@gmail.com](mailto:ngamjitk@gmail.com), Tel +(66)2-256-4142

**Supplementary Material**

**Exclusion criteria for HCs**

HCs were excluded if they had pinguecula or any corneal/conjunctival pathology, had used any topical medication other than artificial tears within the past 3 months, had used drugs that may interfere with tear production (i.e., antidepressants, anticholinergics, antihistamines, antihypertensives, calcium-channel blockers, antacids, systemic corticosteroids, or retinoids) within the past 3 months, or had previous ocular surgery or contact lens use within the past 6 months. Pregnant and breastfeeding patients were excluded.

**Ocular and urine sample collection for HPV detection**

After topically instilling anesthetic eye drops, a sterile FLOQSwab was passed and rolled over the pterygium or conjunctiva repeatedly to collect an adequate number of cells. Afterwards, the swabs were placed in 1.5-mL tubes, and 500 µL of phosphate-buffered saline (PBS) was added. The swabs were then vortexed, and residual samples were transferred to 1.5-mL tubes and stored at 4°C until DNA extraction.

First-void urine (FVU) samples were self-collected in a sterile Colli-Pee device, stored at 4°C, and processed within 3 days. Approximately 10 mL of FVU was then centrifuged at 3,000 rpm for 10 min, then the supernatant was discarded, and the pellet was resuspended in 1 mL of the remaining urine.

DNA was extracted from the residual 1 mL of urine and 500 µL of the eye swab samples in PBS using MagDEA Dx reagents and processed as per the manufacturer’s recommendations (Precision System Science Co., Ltd., Chiba, Japan). The DNA was stored at −20°C until testing.

**HPV detection**

The Anyplex II HPV28 simultaneously identified 19 high-risk (16, 18, 26, 31, 33, 35, 39, 45, 51, 52, 53, 56, 58, 59, 66, 68, 69, 73, and 82) and 9 low-risk (6, 11, 40, 42, 43, 44, 54, 61, and 70) HPVs in two multiplex reactions on the CFX96 real-time thermocycler (Bio-Rad, Hercules, CA, USA). The L1 gene and internal control (human β-globin gene) were amplified concurrently, and the fluorescence was measured continuously with increasing temperature. An internal control was included to check the entire process from DNA extraction to PCR amplification. A negative control and three positive controls provided by the manufacturer were included in each PCR run as requested. Data recording and interpretation were automated using Seegene viewer software (Seegene, Seoul, South Korea) as per the manufacturer’s instructions.
